# Non-suicidal self-injury and suicidal ideation among Chinese college students of childhood emotional abuse: associations with rumination, experiential avoidance, and depression

**DOI:** 10.3389/fpsyt.2023.1232884

**Published:** 2023-08-01

**Authors:** Wan Wang, Xi Wang, Guiqin Duan

**Affiliations:** ^1^Department of Child Development and Behavior, Third Affiliated Hospital of Zhengzhou University, Zhengzhou, China; ^2^College of Computer Science and Electronic Engineering, Hunan University, Changsha, China

**Keywords:** childhood emotional abuse, non-suicidal self-injury, suicidal ideation, rumination, experiential avoidance, depression

## Abstract

**Background:**

Prior studies have proved the relationships between childhood emotional abuse (CEA) histories and suicidal thoughts or behaviors in adulthood, however, how emotion regulation strategies work as the mediating mechanism is necessary to be investigated. This study aimed to further verify the impacts of rumination, experiential avoidance (EA) and depression on the associations between CEA and non-suicidal self-injury (NSSI) and suicidal ideation (SI) on a sample of Chinese college students.

**Methods:**

The Childhood Emotional Abuse Questionnaire, the Non-Suicidal Self-Injury Questionnaire, the Symptom Checklist, the Ruminative Response Scale, the Acceptance and Action Questionnaire-II and the Zung Self-Rating Depression Scale were completed by 1,317 college students.

**Results:**

The rates of NSSI and SI of students with CEA experiences were 31.70 and 7.90% respectively, both higher than those without such experiences. The mediating roles of rumination, EA and depression between CEA and NSSI and SI were significant (*p* < 0.01).

**Conclusion:**

The current study shed light on the linking roles of rumination, EA and depression in the relations between CEA and NSSI and SI. It is suggested that developing adaptive emotion-regulating strategies may be helpful to the intervention of suicidal thoughts or behaviors among individuals with CEA experiences.

## 1. Introduction

Childhood emotional abuse (CEA) refers to long-term negative attitudes towards children by their caregivers, which is a type of psychological, rather than physical, form (1). It involves verbal aggression on one’s sense of value or well-being, and a series of behaviors of criticism, threats, blame, shaming, humbling or demeaning (2). Experiencing emotional abuse during childhood may lead to adverse psychological outcomes that can continue until adulthood (3). Studies focusing on negative influence of childhood adversities have proved that people being emotionally abused as children were more prone to become depressive or anxious (2). Furthermore, CEA has been proved to be linked to suicide thoughts or attempts in adolescence and adulthood (4, 5). Although growing empirical evidence has identified adverse childhood experience related to suicide-related thoughts or behaviors (6, 7), most researches have focused on the negative outcomes of physical or sexual abuse in childhood (8), and few studies have explored the unique influence of CEA on psychological health status. Besides, the underlying psychological mechanism linking the associations between CEA and suicidal thoughts or behaviors remains unclear. Therefore, it is necessary to more exactly clarify the underlying influence process of CEA on suicide-related thoughts or behaviors to effectively prevent the occurrence of adverse events.

Non-suicidal self-injury (NSSI) is the deliberate hurt to body with no death desires, such as hitting or cutting skin (9). Suicidal ideation (SI) refers to excessive brooding about meaninglessness of life, and even thinking of ending life (10). SI and NSSI are associated with each other (11), and people with NSSI behaviors are much likely to experience SI later in their life (12), indicating that they might share similar underlying risk factors (7).

Dysfunctional emotion regulation or maladaptive emotion regulation strategies may be the underlying psychological processes leading to NSSI and SI of CEA individuals. Many studies have regarded childhood abuse as a distal trigger of the maladaptive emotion-regulating strategies (13, 14). Individuals with childhood abuse experiences have deficits or difficulties in regulating their emotions (2, 15), which might further cause subsequent mental health problems (16, 17). For instance, recent empirical evidences suggested that emotion dysregulation works as a crucial mediating factor between CEA and suicidal thoughts or behaviors (18, 19). Therefore, how emotion regulation strategies work as the mediating mechanism between CEA and NSSI and SI is necessary to be further investigated.

As a maladaptive emotion regulation strategy, rumination is an significant risk factor for negative psychological outcomes. Rumination refers to recurring and intrusive thoughts about one’s suffering, its probable reasons, process and consequences of unpleasant feelings (20). It is common among adults who have experienced childhood adverse events (21). Unlike other kinds of strategies bringing up positive problem-solving, rumination is repetitive negative thinking and remains focused on negative experiences and emotions instead of taking action (22), and this strategy could result in adverse psychological outcomes and maladaptive thoughts or behaviors including SI and NSSI (23–25). However, few have demonstrated the mediating effects of rumination between CEA and SI and NSSI behaviors among Chinese college students (5, 26).

Instead of repetitively focusing on their distress and its reasons and outcomes, individuals may also tend to avoid or suppress their negative thoughts and moods. Experiential avoidance (EA) is an intent to avoid or run away from undesirable feelings or environment (27). It is characterized by unwillingness to get in touch with adverse experience and by taking action to suppress traumatic-related distress, urges, or emotions (16). People who have a history of abuse as children tend to adopt EA as an emotional regulation strategy to distract themselves from negative feelings or unwanted emotional experiences (13, 28). However, EA may cause people having more possibilities of psychopathology and higher risks of comorbid psychological outcomes such as NSSI and SI (29–31). Although a prior study on people with childhood sexual abuse histories suggested that EA was positively associated with NSSI and SI, few studies have explored EA’s effect on the relation between CEA and NSSI and SI. Therefore, whether EA might be likely linking the relation between CEA and NSSI and SI remains worth studying.

Consequently, the present study was designed to explore the impacts of CEA on NSSI and SI through rumination, EA and depression among Chinese college students. Based on the emotion regulation models and existing studies, it is hypothesized that Chinese college students emotionally abused as children might be more likely to engage in NSSI behaviors and have SI. Furthermore, we also hypothesized that rumination, EA and depression might be the linking factors in the association between CEA and NSSI and SI.

## 2. Methods

### 2.1. Samples

Thousand three hundred seventeen Chinese college students were recruited online in this study. The effective sample used for statistical analysis in final comprised 1,254 participants ((583 males, 617 females), mean age = 21.16; SD = 3.56), with an effective ratio of 95.22%. Among them, there were 619 (49.36%) students from cities and 635 (50.64%) students from the countryside.

### 2.2. Measures

#### 2.2.1. The childhood emotional abuse questionnaire

The history of CEA was measured by the childhood emotional abuse questionnaire (CEAQ), a subscales of the Childhood Trauma Questionnaire–Short Form [CTQS-SF; (1)]. It contains 5 items assessing emotional abuse histories in childhood, and each item is rated on a 5-point scale (1 = never true, 5 = very often true). Participants with 5 or more scores could be classified as people with a history of CEA. The CEAQ has demonstrated excellent properties in prior researches (32, 33).

#### 2.2.2. The non-suicidal self-injury questionnaire

It lists a series of NSSI behaviors, such as hitting, scratching, pinching, burning, banging head, pulling hair, biting, and cutting (34). Respondents were required to answer if they ever hurt their body intentionally without suicide desire and how many times they conducted each behavior. The frequencies of all behaviors were counted as the total score. This questionnaire utilized in prior research showed satisfactory properties (35).

#### 2.2.3. Suicidal ideation

Suicidal ideation (SI) was assessed with one item ‘I want to end my life,’ rated for frequency on a 5-point Likert-type format (1 = never; 5 = always). Respondents with 2 or more scores could be regarded as having suicidal thoughts. Prior researches have adopted this question to assess SI effectively among different samples (36, 37).

#### 2.2.4. The ruminative response scale

The ruminative response scale (RRS) is a 22-item self-report measurement that aims to evaluate rumination (20). It is consist of three factors: symptom rumination, brooding and reflective pondering (38). Participants have to respond on a 4-point Likert-scale (1 = almost never; 7 = almost always). Many studies have proved the RRS has satisfactory properties (25, 39).

#### 2.2.5. The acceptance and action questionnaire-II

Experiential avoidance was evaluated by the acceptance and action questionnaire-II (AAQ-II) (40), which was revised into a Chinese version (41). The AAQ-II is a 7-item self-assessment scale rating from 1 (never true) to 7 (always true). Many researches have proved its good validity and reliability among different samples (42).

#### 2.2.6. The Zung self-rating depression scale

Depression is measured by the Zung SDS, which is a self-assessment instrument designed to measure a variety of depressive symptoms (43). The Zung SDS contains 20 items rated on a 4-point Likert scale (1 = none, or a little of the time; 4 = most, or all of the time). Several items are reverse scored and the total scores are between 20 and 80. Respondents are instructed to answer each item according to their experiences over the last week. With its satisfactory properties, the Zung SDS has been widely used for the assessment of depression (44).

### 2.3. Procedure

This research conformed to all ethical standards in the Declaration of Helsinki. The participants were recruited by means of online advertisement, mostly through a web-based platform.^1^ Before starting the online survey, they were informed about the aim of scientific research, the confidentiality of the survey, and their rights. The participants received some money in turn for their participation. The Ethics Committee for Scientific Research of the Third Affiliated Hospital of Zhengzhou University approved this research (Grant No. 2022-360-01).

### 2.4. Statistical analysis

All statistical analyses were performed by SPSS 21.0 Software. The independent two-sample *t-*test was conducted to compare the difference between two groups. The relationships between variables were explored by computing the zero-order Pearson’s correlation. The PROCESS macro (model 6) was utilized to validate the effects of CEA on NSSI or SI through rumination, EA and depression by bias-corrected bootstrap estimates based on 2,000 samples (45). The effect was significant with the 95% bootstrap confidence interval not including zero. AMOS 21.0 was utilized to conduct the structural equation model. The theoretical model is well fitted with CMIN/DF < 2, GFI and CFI > 0.95, AGFI>0.90, and RMSEA<0.05 (46, 47).

## 3. Results

### 3.1. Group comparisons

There are 296 (23.60%) students reported NSSI behaviors and 59 (4.70%) reported SI. Compared to students without CEA experiences, those who have such experiences showed higher rates in SI and NSSI (7.9% vs. 1.44, 31.70% vs. 15.41%). Males reported higher rates in SI and NSSI than females (5.00% vs. 4.47, 30.36% vs. 17.73% for SI and NSSI respectively).

Table 1 showed that students with CEA histories showed higher level of rumination, EA, depression, SI and NSSI behaviors (*p* < 0.01). Compared to females, male students reported more CEA experience and NSSI (*p* < 0.01). Besides, students from cities reported more EA scores than those from the countryside (*p* < 0.01).

**Table 1:** Comparison of group means on variables of interest.

	CEA experience			Gender			Birthplace		
	Yes (*n* = 631)	No (*n* = 623)	*t*	Male (*n* = 583)	Female (*n* = 671)	*t*	City (*n* = 619)	Countryside (*n* = 635)	*t*
CEA				6.68(2.65)	6.32(1.97)	2.69^**^	6.53(2.37)	6.45(2.27)	0.30
Rumination	40.05(10.07)	33.02(9.13)	12.94^***^	36.37(11.03)	36.72(9.49)	−0.60	36.77(10.08)	36.35(10.39)	0.72
EA	18.62 (8.90)	13.47 (7.79)	10.89^***^	15.99 (8.97)	16.12 (1.31)	−0.26	16.82 (8.79)	15.32 (8.65)	3.03^**^
Depression	1.40 (0.47)	1.18 (0.31)	9.75^***^	1.27(0.41)	1.31 (0.42)	−1.42	1.30(0.42)	1.28 (0.41)	0.49
NSSI	1.12 (0.45)	1.02 (0.15)	5.17^***^	1.08 (0.39)	1.06 (0.29)	3.22^**^	1.06 (0.34)	1.07 (0.34)	−0.24
SI	1.44 (5.48)	0.69(4.21)	2.72^**^	1.54 (6.64)	0.65(2.50)	0.99	0.85 (2.90)	1.28(6.26)	−1.54

AT, autistic traits; AB, aggressive behaviors. ^**^p < 0.01 and ^***^p < 0.001.

### 3.2. Correlations

Table 2 shows correlations between CEA, rumination, EA, depression, NSSI, SI, age, gender and birthplace. The results indicated that CEA, rumination, EA, depression, NSSI and SI had positive correlations with each other (*r* = 0.14 ~ 0.65, *p* < 0.01). Age was negatively related to CEA, rumination, EA, depression, NSSI and SI (*r* = −0.18 ~ −0.08, *p* < 0.01). Gender had negative correlations with CEA and NSSI (*r* = −0.09 ~ −0.08, *p* < 0.01). Birthplace was negatively related to EA (*r* = −0.09, *p* < 0.01).

**Table 2:** Bivariate correlations between variables (*n* = 1,254).

	1	2	3	4	5	6	7
1. CEA	——						
2. Rumination	0.38**	——					
3. EA	0.28**	0.65**	——				
4. Depression	0.35**	0.56**	0.56**	——			
5. NSSI	0.14**	0.21**	0.20**	0.29**	——		
6. SI	0.25**	0.29**	0.29**	0.56**	0.34**	——	
7. Age	−0.08**	−0.15**	−0.18**	−0.15**	−0.13**	−0.09**	——
8. Gender	−0.08**	0.02	0.01	0.04	−0.09**	−0.03	——
9. Birthplace	−0.02	−0.02	−0.09**	−0.01	0.04	0.01	——
*M*	6.49	36.56	16.06	1.29	1.07	1.07	21.16
*SD*	2.32	10.24	8.75	0.41	4.90	0.34	3.56

CEA, childhood emotional abuse; EA, experiential avoidance; NSSI, non-suicidal self-injury; SI, suicidal ideation. **p < 0.01 and ***p < 0.001. 1 = male, 2 = female.

### 3.3. Direct and indirect effects

The effects of CEA on NSSI through rumination, EA and depression were examined by PROCESS, controlling for age, gender and birthplace (Table 3). The results showed the indirect effect of CEA on NSSI through rumination was significant (*β* = 0.07, *p* < 0.01), as was its indirect effect through rumination and depression (*β* = 0.38, *p* < 0.01; *β* = 0.62, *p* < 0.001; *β* = 0.24, *p* < 0.001). The indirect effect of CEA on NSSI through rumination, EA and depression also was significant (*β* = 0.38, *p* < 0.01; *β* = 0.62, *p* < 0.001; *β* = 0.34, *p* < 0.001; *β* = 0.24, p < 0.001). But the direct effects of CEA on EA and NSSI were not significant (*β* = 0.03, *p* = 0.14; *β* = 0.02, *p* = 0.43).

**Table 3:** Effects of childhood emotional abuse on non-suicidal self-injury via rumination, experiential avoidance and depression.

Models	Dependent variable	Independent variable	*β*	SE	LL 95% CI	UL 95% CI	*R* ^2^	*F*
Model 1	Rumination	CEA	0.38**	0.19	0.01	0.24	0.17	60.78***
Model 2	EA	CEA	0.03	0.02	−0.01	0.08	0.43	186.05***
		Rumination	0.62***	0.02	0.57	0.66		
Model 3	Depression	CEA	0.15***	0.02	0.10	0.20	0.40	137.61***
		Rumination	0.28***	0.03	0.22	0.34		
		EA	0.34***	0.03	0.28	0.39		
Model 4	NSSI	CEA	0.02	0.03	−0.04	0.08	0.11	20.48***
		Rumination	0.07**	0.04	0.03	0.12		
		EA	0.02	0.04	−0.06	0.39		
		Depression	0.24***	0.04	0.17	0.34		

CEA, childhood emotional abuse; EA, experiential avoidance; NSSI, non-suicidal self-injury. **p < 0.01 and ***p < 0.001.

To clarify the effects of CEA on SI through rumination, EA and depression, after controlling for gender, age and birthplace, another model was conducted. As shown in Table 4, the direct effect of CEA on SI was significant (*β* = 0.08, *p* < 0.01), as was its indirect effect through rumination and depression (*β* = 0.38, *p* < 0.01; *β* = 0.62, *p* < 0.001; *β* = 0.58, *p* < 0.001). Besides, the indirect effect of CEA on SI via rumination, EA and depression also was significant (*β* = 0.38, *p* < 0.01; *β* = 0.62, *p* < 0.001; *β* = 0.34, *p* < 0.001; *β* = 0.58, *p* < 0.001). But, the direct effects of CEA on EA, EA on SI, and rumination on SI were non-significant (*β* = 0.03, *p* = 0.14; *β* = −0.02, *p* = 0.58; *β* = −0.05, *p* = 0.17).

**Table 4:** Effects of childhood emotional abuse on suicidal ideation via rumination, experiential avoidance and depression.

Models	Dependent variable	Independent variable	*β*	SE	LL 95% CI	UL 95% CI	R^2^	F
Model 5	Rumination	CEA	0.38**	0.03	0.32	0.43	0.17	60.78***
Model 6	EA	CEA	0.03	0.02	−0.01	0.08	0.43	186.05***
		Rumination	0.62***	0.02	0.57	0.66		
Model 7	Depression	CEA	0.15***	0.02	0.10	0.20	0.40	137.61***
		Rumination	0.28***	0.03	0.22	0.34		
		EA	0.34***	0.03	0.28	0.39		
Model 8	SI	CEA	0.08**	0.03	0.02	0.13	0.33	84.16***
		Rumination	−0.05	0.03	−0.11	0.02		
		EA	−0.02	0.03	−0.08	0.05		
		Depression	0.58***	0.03	0.52	0.64		

CEA, childhood emotional abuse; EA, experiential avoidance; SI, suicidal ideation. **p < 0.01 and ***p < 0.001.

**Figure 1:**
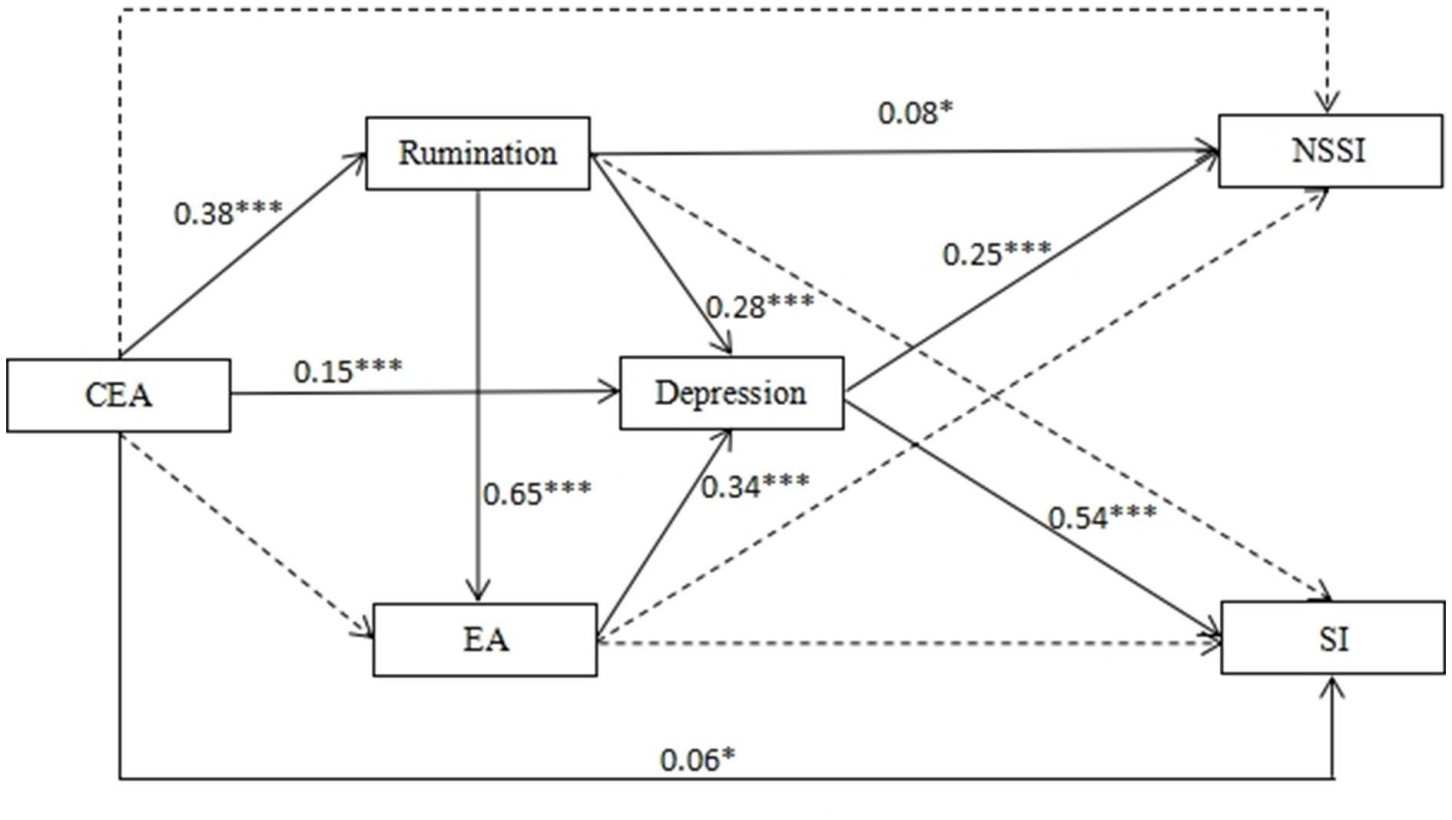
The total model. All coefficient for paths in the structural equation model were standardized CEA, childhood emotional abuse; EA, experiential avoidance; NSSI, non-suicidal self-injury; SI, suicidal ideation. *p < 0.05; **p < 0.01; ***p < 0.001.

To further visualize the effects of CEA on NSSI and SI through rumination, EA and depression, a total model was performed. The indices indicated a satisfactory fit for the data (CMIN/DF = 1.628, GFI = 0.996, CFI = 0.999, AGFI = 0.996, RMSEA = 0.022). The standardized path coefficients of the total model are shown in Figure 1.

## 4. Discussion

Our study found that males reported higher rates in SI and NSSI than females. However, previous studies showed no difference in rates of NSSI and SI between males and females, or that NSSI and SI were more common among females (48, 49). These discrepancies might caused by differences in samples, methods of measurement and particular definitions of NSSI and SI (50). In addition, our study demonstrated that high prevalence of NSSI was related to a significant risk of suicide. On the basis of the Three-Step Theory of Suicide, suicide capacity facilitates the transition from SI to suicide attempt (51). NSSI behaviors help to develop one’s abilities to overcome fear of death and access lethal means, which are risk factors for committing suicide (52).

This study explored the impacts of CEA on NSSI and SI through rumination, EA and depression among Chinese college students. An important finding of our study is 50.32% students had CEA experiences, of whom there were 7.9% reported SI and 31.70% reported NSSI behaviors, both higher than those without such experiences. There were positive associations between CEA and NSSI and SI, and students with CEA experiences reported more NSSI behaviors and SI. This result was consistent with prior researches (5, 53), suggesting that those emotionally abused as children had more vulnerability for suicidal thoughts or behaviors. Many studies have also regarded childhood trauma as a significant risk factor that could lead to psychological problems in adulthood (2, 13).

In line with prior studies, our study demonstrated that CEA was positively related to rumination and EA, which are maladaptive emotion regulation strategies (21, 26, 28). The ability of regulating emotion is developed in the circumstances of interpersonal emotional communication between caregivers and children at the early stage of life (54), but childhood trauma, especially repeated emotional abuse imposed on children by caregivers, hinders the learning of effective emotion-regulating skills (55). People with childhood abuse histories are more prone to have fewer adaptive emotion-regulating skills and more deficits or difficulties in identifying, understanding, and expressing their feelings (17).

As expected, our study also confirmed the mediating roles of rumination, EA and depression on the associations between CEA and NSSI and SI, indicating that maladaptive emotion-regulating strategies may be the underlying psychological processes leading to NSSI and SI of CEA individuals. These finding extends the literature by supporting the notion that emotion dysregulation plays a crucial mediating effect in the associations between CEA and suicidal thoughts or behaviors (18, 19). First, rumination is one linking factor in the relation between CEA and NSSI and SI. People who have been emotionally abused during childhood tend to ruminatively thinking their negative emotions, feelings, reasons and consequences of the traumatic events (21). On the basis of the Response Styles Theory, rumination is a risk factor for individuals’ mental health status (22). Instead of taking action to solve problems, these repetitive negative thoughts and emotion regulation style may result in more severe psychological outcomes and maladaptive thoughts or behaviors including depression, SI and NSSI (23, 24). Second, EA is another linking factor in the relations between CEA and NSSI and SI. For people with CEA experiences, EA is an emotional regulation strategy to distract themselves from negative emotions or unpleasant internal feelings or external circumstance (13, 28). However, EA could not only help people to shake off traumatic-related distress thoughts and emotions briefly but also in turn increases the risks of depression, NSSI behaviors and SI (56). Besides, EA following CEA may weaken with one’s ability to effectively solve problems. Therefore, as a maladaptive emotion-regulating strategy, EA might cause CEA individuals being more vulnerable to suicide-related thoughts and behaviors (29, 31).

### 4.1. Limitations

This study has some limitations needed to be pointed out. First of all, because of its cross-sectional design, this study cannot draw causal conclusions. So it’s necessary to conduct longitudinal-design studies in the future to verify the findings of our research. Second, the way of self-evaluation and retrospective assessment may bring about inaccuracy in evaluating variables. Third, our study focused on CEA rather than other kinds of childhood trauma like physical abuse. It will be meaningful to explore other kinds of childhood trauma and compare their influence with that of CEA in subsequent researches. Finally, there might be bias in the responses due to social desirability (e.g., Suicidal Ideation, the Non-Suicidal Self-Injury Questionnaire), which could be reduced by maintaining anonymity of questionnaires.

## 5. Conclusion

In summary, this study demonstrates the impacts of CEA on NSSI and SI through rumination, EA and depression. These findings could help us further clarify of the relations between CEA, rumination, EA, depression, NSSI and SI. Therefore, subsequent studies are needed to verify if developing effective emotion-regulating strategies could be helpful to the intervention of suicidal thoughts or behaviors among people with emotional abuse experience in childhood.

## Data availability statement

The raw data supporting the conclusions of this article will be made available by the authors, without undue reservation.

## Ethics statement

The studies involving human participants were reviewed and approved by the Ethics Committee for Scientific Research of the Third Affiliated Hospital of Zhengzhou University. The patients/participants provided their written informed consent to participate in this study.

## Author contributions

WW made substantial contributions to the design of the work and wrote the manuscript. XW and GD did a lot of work in data collecting, statistics analysis, and revision of the manuscript. All the authors agree to be accountable for all aspects of the work in ensuring that questions related to the accuracy or integrity of any part of work are appropriately investigated and resolved.

## Funding

This work was supported by the PhD Research Startup Foundation of Third Affiliated Hospital of Zhengzhou University (Grant Number: BS20230106).

## Conflict of interest

The authors declare that the research was conducted in the absence of any commercial or financial relationships that could be construed as a potential conflict of interest.

## Publisher’s note

All claims expressed in this article are solely those of the authors and do not necessarily represent those of their affiliated organizations, or those of the publisher, the editors and the reviewers. Any product that may be evaluated in this article, or claim that may be made by its manufacturer, is not guaranteed or endorsed by the publisher.

## Footnotes

^1^ https://www.wjx.cn/
